# Five-dimensional tracking of single nanoparticles in living cells

**DOI:** 10.1038/s41377-018-0026-9

**Published:** 2018-06-06

**Authors:** Xiangzhao Ai, Bengang Xing

**Affiliations:** Division of Chemistry and Biological Chemistry, School of Physical & Mathematical Sciences, Nanyang Technological University, Singapore, 637371 Singapore

**An innovative approach to achieve dynamic five-dimensional tracking of single nanoparticles under simple microscopic inspection by human eyes was presented; this approach provided an effective means to investigate the complicated biological microenvironment in living cells.**

Currently, owing to the ongoing development of single-molecule techniques over the past few decades, it is possible to explore the underlying mechanisms of diverse biological processes in living cells, such as dynamically monitoring cell membrane behaviors, particularly the stepping of motor proteins along microtubules, and spatiotemporally scanning gene expression or regulation in nuclei^1^. Effective inspection and tracking of target-specific imaging probes provide unprecedented abilities for researchers to directly observe the biological activities of various cellular components (e.g., organelles, enzymes, ions, etc.) at the single-molecule level with ultimate three-dimensional (3D) precision and time resolution. A number of optical contrast agents, including organic chromophores and inorganic materials, have been widely applied in these studies^2^. However, critical challenges still remain in regard to eliminating the undesired drawbacks of the commonly used probes during long-term imaging periods, including photobleaching and degradation of organic dyes, photoblinking and toxicity of quantum dots, and inherent autofluorescence in living organisms^3^. Therefore, development of novel materials that allow strong emissions, high photostabilities, lower cytotoxicities, and limited backgrounds is highly desirable.

Recently, lanthanide-doped upconversion nanoparticles (UCNPs) have attracted much attention in materials science due to their distinctive optical properties, which enable them to convert long-wavelength near-infrared (NIR) light into multiplexed short-wavelength emissions that span a broad range, from the ultraviolet/visible to the NIR region^4^. UCNPs are considered to be suitable probes for long-term tracking in imaging processes, mainly due to their promising advantages, such as non-bleaching, non-blinking, autofluorescence-free, low cytotoxicity, and high chemical/photo-stability. Despite these remarkable optical capabilities, current studies on UCNPs utilizing conventional far-field fluorescence microscopy are limited because their resolution is insufficient to distinguish a single nanoparticle from clusters or aggregates, which is significant for real-time observations of sub-cellular component movements to understand their potential functions in complex cellular environments^5^. Thus, innovative approaches are still in high demand to determine the activities of individual UCNP during long-term imaging periods in biological investigations.

**Fig. 1:**
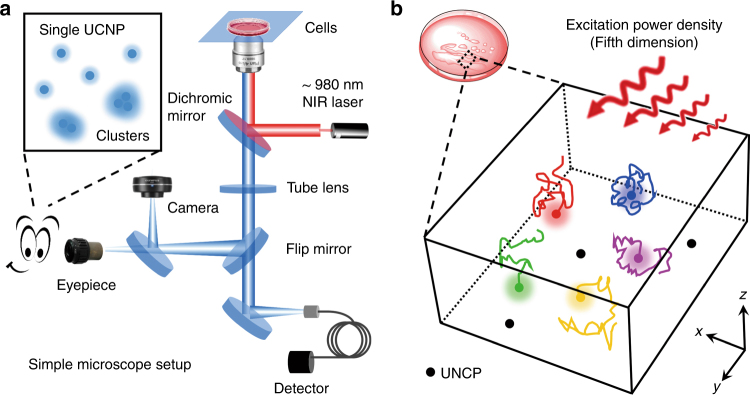
5D tracking of a single UCNP in living cells. **a** The simple microscopic system for the inspection of a single nanoparticle with the human eye to distinguish individual particles from clusters. **b** The trajectories of a particular UCNP with a tunable emission upon different power intensity excitation (the fifth dimension) to investigate the complicated intracellular microenvironment

For this purpose, in a recent publication, Jin and co-workers demonstrated that the human eye could directly distinguish single UCNPs and identify relevant information, including their color, intensity, and location in cells, under a simple microscope setup (Fig. 1a)^6^. Interestingly, these authors determined that at least 4186 photons per 100 ms are required for our eyes to differentiate two separate blue UCNPs upon ~980 nm NIR light excitation. By utilizing a series of size- and emission-tunable UCNPs, they achieved long-term quantitative analysis of one nanoparticle with bright, uniform, and stable upconverted luminescence signals in living cells. Remarkably, it is easy and accurate for researchers to recognize clusters or aggregates of UCNPs when the emitters present a brightness higher than the threshold value during microscopic inspection.

Inspired by these promising results, the authors further investigated the heterogeneous diffusion dynamics of each UCNP and cluster in the different intracellular microenvironment. Interestingly, it was indicated that the diffusion coefficient of a single nanoparticle in early endosomes (0.18 μm^2^/s) was slightly larger than that in late endosomes/lysosomes (0.04 μm^2^/s), and the relatively faster movement of UCNP with a specific direction (3 μm/s) was associated with the active migration of molecular motor proteins on microtubules or actin filaments. Moreover, they also calculated the localized environment viscosity of each particle or cluster, which provided powerful insight into their surrounding protein dynamics in living cells. Compared with the value in the cytoplasm (11.6 ± 0.9 cP), the viscosity in early endosomes (41 ± 4 cP) was obviously lower than that in late endosomes/lysosomes (184 ± 14 cP), suggesting the improved protein functional capacities in the intracellular cytosol and early endosomes. More importantly, besides the 3D position and multiple color domain of each UCNP, Jin and co-workers proposed that the excitation power density could act as the fifth dimension to simultaneously monitor various nanoparticles due to their power-dependent optical properties (Fig. 1b). By adjusting the suitable concentrations of doped ions, a single UCNP could be lit up using different irradiation densities, which revealed the specific superiority of an upconversion fluorescence microscope for bioimaging studies.

In summary, this work presented a novel approach to achieve dynamic tracking of individual UCNPs based on unique 5D imaging modalities in living cells. These capabilities are extremely significant for long-term imaging of biological systems, which will not only allow the human eye to directly identify information about particular nanoparticles via a simple microscopic inspection but also initiate an effective method to investigate the complicated biological microenvironment by utilizing a single-particle technique in physiologic and pathologic processes in living conditions.
